# Performance of an Idiopathic Pulmonary Fibrosis–Derived Multibiomarker Panel for Rheumatoid Arthritis–Associated Interstitial Lung Disease

**DOI:** 10.1002/art.43383

**Published:** 2026-01-11

**Authors:** Brent A. Luedders, Daniel Kass, Joshua F. Baker, Michael J. Duryee, Yangyuna Yang, Punyasha Roul, Halie Frideres, Katherine D. Wysham, Paul A. Monach, Andreas Reimold, Gail S. Kerr, Gary Kunkel, Grant W. Cannon, Scott M. Matson, Jill A. Poole, Geoffrey M. Thiele, Ted R. Mikuls, Bryant R. England, Dana P. Ascherman

**Affiliations:** ^1^ VA Nebraska‐Western Iowa Health Care System Omaha; ^2^ University of Nebraska Medical Center Omaha; ^3^ University of Pittsburgh Pittsburgh Pennsylvania; ^4^ Corporal Michael J. Crescenz VA Medical Center Philadelphia Pennsylvania; ^5^ University of Pennsylvania Philadelphia; ^6^ VA Puget Sound Health Care System Seattle Washington; ^7^ University of Washington Seattle; ^8^ VA Boston Health Care System Boston Massachusetts; ^9^ VA North Texas Health Care System Dallas; ^10^ University of Texas Southwestern Dallas; ^11^ Washington VA Medical Center Washington DC; ^12^ Howard University Washington DC; ^13^ Georgetown University Washington DC; ^14^ Salt Lake City VA Health Care System Salt Lake City Utah; ^15^ University of Utah Salt Lake City; ^16^ University of Kansas Kansas City

## Abstract

**Objective:**

To assess whether a panel of peripheral blood biomarkers associated with idiopathic pulmonary fibrosis (IPF) is also associated with interstitial lung disease (ILD) in patients with rheumatoid arthritis (RA) using three independent cohorts.

**Methods:**

We first assessed the association of a panel of IPF‐associated biomarkers with prevalent ILD among two separate RA cohorts (n = 93 and n = 71). Concentrations of eight IPF‐related biomarkers (eotaxin, Flt‐3L, interleukin‐8, macrophage‐derived chemokine, monocyte chemoattractant protein 1, and matrix metalloproteinase 2/7/9 [MMP‐2/7/9]) were measured, standardized, and summed to generate a multibiomarker score. We subsequently validated the association of this score (minus MMP‐2) with prevalent and incident ILD in an independent multicenter prospective cohort of US veterans with RA (n = 2,507). Multivariable regression models were adjusted for relevant covariates in the validation cohort.

**Results:**

In both development cohorts, participants with RA‐ILD had significantly higher IPF multibiomarker scores than those with RA alone. In the independent validation cohort, participants in the highest quartile of multibiomarker scores had a significantly higher likelihood of prevalent ILD (adjusted odds ratio 2.14 [95% confidence interval 1.18–3.87]) and incident ILD (adjusted hazard ratio 2.45 [95% confidence interval 1.55–3.88]) than those in the lowest quartile. The cumulative hazard of incident ILD approached 20% by 15 years for those in the highest quartile, compared to <10% for all other quartiles.

**Conclusion:**

A multibiomarker panel derived from IPF‐associated biomarkers was associated with RA‐ILD in separate development and validation cohorts. This overlap supports the concept of shared etiopathogenesis of IPF and RA‐ILD and illustrates the potential for peripheral blood biomarker panels to stratify ILD risk among patients with RA.

## INTRODUCTION

Interstitial lung disease (ILD) is a common extra‐articular manifestation of rheumatoid arthritis (RA), with clinically relevant RA‐ILD identified in approximately 5% to 15% of people with RA.^1^, ^2^, ^3^ The prognosis after the identification of RA‐ILD is poor, with most studies showing a median survival of approximately three to eight years.^4^ Given this poor prognosis, strategies to identify RA‐ILD at earlier stages are needed. The importance of early identification of ILD is reflected in recent guidelines from the American College of Rheumatology (ACR)/American College of Chest Physicians,^5^ which recommend screening for ILD in patients with RA at increased risk for ILD. However, guidance is limited on which patients warrant screening and how often screening should be completed. A number of clinical risk factors for RA‐ILD have been identified, including cigarette smoking, male sex, older age, and higher articular disease activity,^6^ but these clinical risk factors alone are inadequate to accurately identify RA‐ILD.^7^ Therefore, strategies to more precisely risk‐stratify individuals at risk for RA‐ILD are needed to help inform individual patient monitoring and treatment, as well as to identify patients at high risk for or with early RA‐ILD to include in clinical studies.

Identification of peripheral biomarkers associated with RA‐ILD may not only help to inform risk stratification and screening but also further our understanding of the processes leading to the development and/or progression of ILD. Indeed, a number of candidate biomarkers have been evaluated in previous studies.^8^ Many of these were initially studied in idiopathic pulmonary fibrosis (IPF), the most common idiopathic interstitial pneumonia and a disease that shares epidemiologic and genetic risk factors as well as histopathologic and radiologic features with RA‐ILD.^9^ In previous work comparing the levels of 45 protein biomarkers among individuals with RA‐ILD or IPF and healthy controls, the levels of eight were significantly higher in individuals with IPF (eotaxin, Flt‐3L, interleukin‐8 [IL‐8], macrophage‐derived chemokine [MDC], matrix metalloproteinase 2/7/9 [MMP‐2/7/9], and monocyte chemoattractant protein 1 [MCP‐1]).^10^ Moreover, the levels of six of these (eotaxin, Flt‐3L, IL‐8, MMP‐2/7/9) were also increased in individuals with RA‐ILD, demonstrating overlap in peripheral blood biomarker profiles.^10^

The objective of this study was to evaluate the ability of IPF‐derived peripheral biomarkers to risk‐stratify ILD in independent RA cohorts. Due to the shared histopathologic and radiologic features of RA‐ILD and IPF, we hypothesized that a biomarker panel linked to IPF would also be significantly associated with prevalent and incident RA‐ILD risk.

## PATIENTS AND METHODS

### Study design

We initially conducted a cross‐sectional study to derive a composite multibiomarker score for RA‐ILD using two cohorts of patients with RA with and without ILD. We subsequently validated the score using an independent multicenter prospective RA cohort, the Veterans Affairs Rheumatoid Arthritis (VARA) Registry. Validation was performed with both cross‐sectional (prevalent ILD) and cohort (incident ILD) study designs. The study was approved by the participating centers’ institutional review boards and by the VARA Scientific Ethics Advisory Committee. All study participants provided informed consent.

### Analysis of IPF multibiomarker score

For initial assessment of the IPF multibiomarker score, we used two separate cohorts of patients with RA with and without ILD. Selection of these cohorts has been previously described in detail.^10^, ^11^ Briefly, individuals meeting the 1987 ACR RA classification criteria^12^ were prospectively enrolled in separate Veterans Affairs (VA) (n = 103) and non‐VA (n = 71) cohorts. Participants in the VA cohort were recruited prospectively from three VA facilities (Miami, FL; Washington, DC; and Dallas/North Texas; n = 53) or were enrolled in the VARA Registry (n = 50). Participants from the non‐VA cohort were recruited from the University of Pittsburgh and Brigham and Women's Hospital. Individuals were classified as RA without ILD, RA with subclinical ILD, or RA‐ILD based on a combination of clinical features, pulmonary function testing, and high‐resolution computed tomography (HRCT) findings that included ground glass opacification, septal thickening, subpleural reticulation, honeycombing, and/or traction bronchiectasis. Chest HRCT scans were assessed by radiologists blinded to the clinical data. Those with RA‐ILD were further classified as having a usual interstitial pneumonia (UIP) or non‐UIP pattern based on the radiologists’ determination.

Based on their association with IPF in previous work,^10^ we measured concentrations of eotaxin, Flt‐3L, IL‐8, MDC, MCP‐1, and MMP‐2/7/9 from serum samples using a Luminex xMAP bead‐based multiplex enzyme‐linked immunosorbent assay (ELISA) platform (EMD Millipore). Concentrations were standardized based on cohort‐specific means and SDs, in which the standardized concentration was equal to the difference between the serum concentration and the cohort mean divided by the SD for each analyte. We then calculated a composite multibiomarker score for each participant by summing the standardized concentrations for individual analytes. MMP‐9 was excluded from the biomarker score in the non‐VA cohort, as 15 of 71 patients had MMP‐9 concentrations above the upper limit of detection, precluding accurate calculation of cohort mean and SD. For each cohort, we performed pairwise comparisons of the multibiomarker score between the RA without ILD, RA with subclinical ILD (imaging evidence of ILD without clinical manifestations of cough or dyspnea), non‐UIP RA‐ILD, and UIP RA‐ILD groups using Mann‐Whitney U tests.

### Validation in VARA cohort

We externally validated this IPF‐derived multibiomarker score in a cohort of US veterans with RA enrolled in the VARA Registry, a multicenter prospective cohort of US veterans with RA.^13^ All participants in the registry meet 1987 ACR criteria for RA.^12^ Clinical data were collected at registry enrollment and longitudinally during clinical follow‐up as per standard of care. RA articular disease activity measured by the Disease Activity Score in 28 joints (DAS28) was calculated at registry enrollment. Serum and plasma samples were collected at the time of registry enrollment and stored at −70°C. Anti–cyclic citrullinated peptide (anti‐CCP) antibody was measured by ELISA using banked serum samples. Registry enrollment began in 2003, and the current study included data collection through December 4, 2023.

For those patients in the VARA Registry, ILD was identified and validated by systematic medical record review as described previously.^14^, ^15^ Registry participants were screened for the presence of RA‐ILD by diagnostic codes and were classified as having RA‐ILD if they were diagnosed with ILD by their treating provider plus had either chest computed tomography (CT) or lung biopsy features consistent with ILD. ILD pattern was classified as UIP if this was either documented in the clinical radiology read or when honeycombing was present and was classified as non‐UIP for all other reads. Prevalent ILD was defined as being present at the time of registry enrollment, and incident ILD defined as developing at any time after registry enrollment during longitudinal follow‐up, with censoring for death or the end of the study period.

Using banked samples collected at registry enrollment, plasma/serum analytes included in the multibiomarker score (eotaxin, Flt‐3L, IL‐8, MDC, MCP‐1, and MMP‐7/9) were measured using R‐PLEX ELISA assays from MesoScale Diagnostics.^15^ MMP‐2 concentrations were not available in the VARA cohort and therefore were excluded from the score. Concentrations of the biomarkers were log‐transformed to normalize their distributions, and the multibiomarker score was calculated as described for the development cohorts using standardized concentrations of the individual analytes. To account for the possibility of nonlinear associations between the multibiomarker score and ILD, we also divided the cohort into quartiles based on the multibiomarker score, with quartile 1 representing the lowest biomarker score values and quartile 4 representing the highest values.

### Statistical analysis

The association of the IPF multibiomarker score with prevalent and incident RA‐ILD in the VARA cohort was assessed using logistic and Cox regression models, respectively, adjusted for age, sex, race, smoking status, anti‐CCP antibody status, and DAS28. For prevalent analyses, participants who developed incident RA‐ILD during longitudinal follow‐up were excluded, and for incident analyses, participants who had prevalent RA‐ILD at baseline were excluded. Discrimination of prevalent RA‐ILD was evaluated with receiver operating characteristic (ROC) curves, initially with clinical predictors alone and subsequently with the addition of the multibiomarker score. In exploratory analyses, we evaluated score performance by assessing the sensitivity, specificity, positive predictive value, and negative predictive value at varying cutoff values. The predictive ability of the multibiomarker score for incident RA‐ILD compared to clinical predictors alone was assessed with Harrell's C statistic.

Beyond these assessments, we also performed exploratory analyses in which we measured the association of the IPF biomarker score with RA‐ILD stratified by radiographic pattern (UIP vs non‐UIP). Due to strong associations of both prevalent and incident RA‐ILD with MMP‐7 concentrations in prior work,^15^ we performed sensitivity analyses in which MMP‐7 was excluded from the multibiomarker score. Additionally, due to overlap of a small number of participants between the VA development cohort and the VARA validation cohort (n = 50), we performed a sensitivity analysis in which these participants were excluded from the regression models in the VARA validation cohort. Due to the possibility of disease latency, we also performed a sensitivity analysis in which the regression models were performed with prevalent ILD defined as ILD identified before or within the first year after registry enrollment and incident ILD defined as developing one or more years after enrollment.

## RESULTS

### Patient characteristics in study cohorts

The development cohorts consisted of 103 participants in the VA cohort and 71 participants in the non‐VA cohort, characteristics of which have been reported previously.^10^ Ten participants were excluded from the original VA cohort due to having incomplete biomarker measurements; therefore, 93 participants from the VA development cohort were included in the current analyses (Supplemental Table 1). In the validation cohort from the VARA Registry, a total of 2,507 participants with complete biomarker measurements were included in analyses. Of these participants, 106 had prevalent ILD and 153 developed incident ILD during 20,142 (mean 8.4 years) patient‐years of follow‐up (Table 1). Seven participants from the VARA Registry were excluded from analyses because available information was indeterminate for the presence/absence of ILD. Compared to participants without ILD, those with prevalent ILD were older, had a longer duration of RA, and were more likely to be anti‐CCP positive and current/former smokers (Table 1).

**Table 1:** Baseline characteristics of participants in the Veterans Affairs Rheumatoid Arthritis Registry (validation cohort)*

	RA without ILD	RA‐ILD	*P* value
Cross‐sectional study of prevalent RA‐ILD			
n	2,241	106	–
Age, mean (SD), y	63.8 (11.2)	68.0 (9.4)	<0.001
Male sex, %	88.0	91.5	0.27
White race, %	77.7	77.4	0.94
Body mass index^a^	29.0	29.2	0.67
Smoking status, %^a^			0.015
Never	21.4	9.8	–
Former	53.8	64.7	–
Current	24.7	25.5	–
RA duration, mean (SD), y^a^	11.2 (11.2)	14.3 (13.8)	0.005
Anti‐CCP positive, %^a^	76.1	85.8	0.021
DAS28, mean (SD)^a^	3.74 (1.57)	4.03 (1.20)	0.07
Multibiomarker score, mean (SD)	−0.08 (3.78)	1.04 (3.99)	0.003
Eotaxin	0.01 (0.99)	0.09 (1.03)	0.34
Flt‐3L	−0.02 (0.97)	0.31 (0.98)	<0.001
IL‐8	−0.01 (0.98)	0.04 (1.01)	0.59
MCP‐1	0.01 (0.97)	−0.01 (1.17)	0.83
MDC	0.02 (0.96)	−0.08 (1.17)	0.29
MMP‐7	−0.04 (0.97)	0.54 (1.27)	<0.001
MMP‐9	−0.04 (1.00)	0.15 (0.99)	0.047
Cohort study of incident RA‐ILD			
n	2,241	153	–
Age, mean (SD), y	63.8 (11.2)	64.6 (9.1)	0.37
Male sex, %	88.0	92.8	0.07
White race, %	77.7	70.6	0.043
Body mass index^b^	29.0	28.5	0.28
Smoking status, %^b^			0.22
Never	21.4	17.1	–
Former	53.8	52.6	–
Current	24.7	30.3	–
RA duration, mean (SD), y^b^	11.2 (11.2)	10.9 (10.8)	0.78
Anti‐CCP positive, %^b^	76.1	83.0	0.052
DAS28, mean (SD)^b^	3.74 (1.57)	4.08 (1.46)	0.010
Multibiomarker score, mean (SD)	−0.08 (3.78)	0.60 (4.08)	0.031
Eotaxin	−0.01 (0.99)	0.06 (1.12)	0.43
Flt‐3L	−0.02 (0.97)	−0.06 (1.16)	0.61
IL‐8	−0.01 (0.98)	0.15 (1.15)	0.048
MCP‐1	0.01 (0.97)	0.04 (1.13)	0.72
MDC	0.02 (0.96)	−0.04 (1.16)	0.45
MMP‐7	−0.04 (0.97)	0.26 (0.94)	<0.001
MMP‐9	−0.04 (1.00)	0.19 (1.00)	0.005

* CCP, cyclic citrullinated peptide; DAS28, Disease Activity Score in 28 joints; IL, interleukin; ILD, interstitial lung disease; MCP, monocyte chemoattractant protein 1; MDC, macrophage‐derived chemokine; MMP, matrix metalloproteinase; RA, rheumatoid arthritis.

^a^ Missing data: body mass index (n = 163 RA without ILD, n = 4 RA‐ILD), smoking status (n = 59 RA without ILD, n = 4 RA‐ILD), RA duration (n = 56 RA without ILD, n = 3 RA‐ILD), anti‐CCP (n = 1 RA without ILD), DAS28 (n = 162 RA without ILD, n = 6 RA‐ILD).

^b^ Missing data: body mass index (n = 163 RA without ILD, n = 3 RA‐ILD), smoking status (n = 59 RA without ILD, n = 1 RA‐ILD), RA duration (n = 56 RA without ILD, n = 3 RA‐ILD), anti‐CCP (n = 1 RA without ILD), DAS28 (n = 162 RA without ILD, n = 4 RA‐ILD).

### IPF multibiomarker score in RA development cohorts

**Figure 1:**
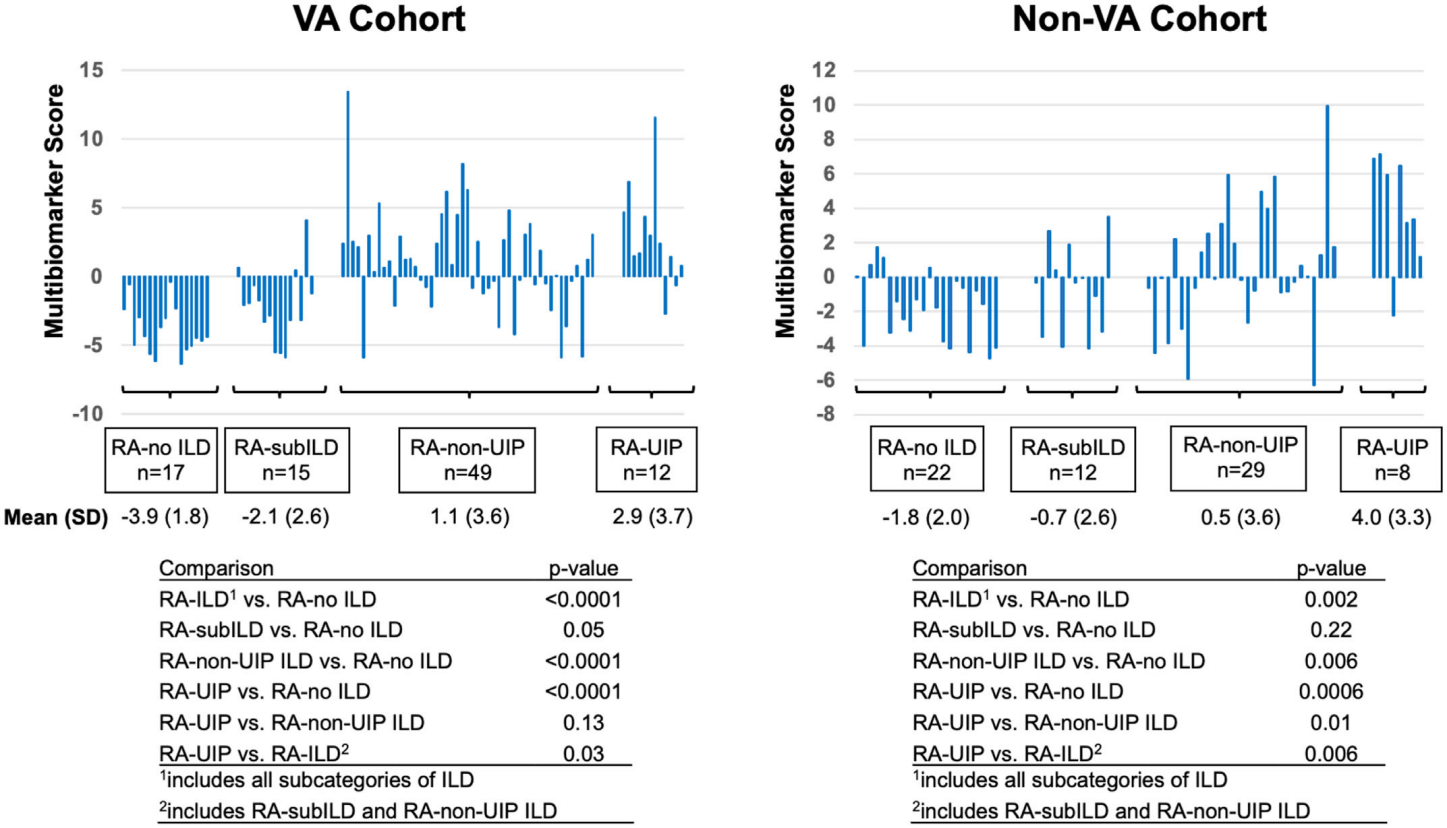
IPF multibiomarker score correlates with stage of RA‐ILD in independent development cohorts. Bar graphs correspond to composite IPF multibiomarker scores in patients with RA without ILD, with subclinical ILD, or with clinically evident ILD (distinguished by the presence vs absence of UIP‐like abnormalities on chest CT). MMP‐9 was excluded from the score in the non‐VA cohort. Tables list *P* values resulting from subgroup comparisons using Mann‐Whitney U tests. CT, computed tomography; ILD, interstitial lung disease; IPF, idiopathic pulmonary fibrosis; MMP, matrix metalloproteinase; RA, rheumatoid arthritis; UIP, usual interstitial pneumonia; VA, Veterans Affairs.

For both the VA and non‐VA development cohorts, patients with RA with ILD had significantly higher IPF multibiomarker scores than those without ILD (mean biomarker scores: VA 1.1 [non‐UIP] and 2.9 [UIP] vs −3.9 [RA alone], *P* < 0.0001; non‐VA 0.5 [non‐UIP] and 4.0 [UIP] vs −1.8 [RA alone], *P* = 0.002; Figure 1). The multibiomarker score was highest among those with a UIP pattern of ILD compared to those with a non‐UIP pattern, though this was only significant in the non‐VA cohort. Multibiomarker scores did not significantly differ between those with subclinical ILD and those without ILD.

### IPF multibiomarker score in RA validation cohort

In the VARA validation cohort, participants with prevalent ILD had higher IPF multibiomarker scores than those without ILD (mean 1.04 vs −0.08; *P* = 0.003). Among individual analytes in the multibiomarker score, Flt‐3L, MMP‐7, and MMP‐9 concentrations were all significantly higher in participants with prevalent ILD (Table 1). In adjusted analyses, higher multibiomarker scores were associated with greater odds of prevalent ILD (adjusted odds ratio [aOR] 1.07 [95% confidence interval (CI) 1.01–1.14] per one‐point increase in multibiomarker score). Those individuals in the highest quartile of IPF multibiomarker scores had greater than two‐fold increased odds of prevalent ILD (aOR 2.14 [95% CI 1.18–3.87]; Table 2) compared to those in the lowest quartile.

**Table 2:** Associations of the IPF multibiomarker score with prevalent and incident RA‐ILD in the VARA validation cohort*

Multibiomarker score quartile	Adjusted odds or hazard ratio (95% CI)^a^	*P* value
Prevalent ILD		
Quartile 1	Ref.	–
Quartile 2	0.74 (0.35–1.55)	0.43
Quartile 3	1.45 (0.78–2.72)	0.24
Quartile 4	2.14 (1.18–3.87)	0.01
*P* trend	–	0.005
Incident ILD		
Quartile 1	Ref.	–
Quartile 2	1.06 (0.63–1.78)	0.83
Quartile 3	1.32 (0.80–2.19)	0.28
Quartile 4	2.45 (1.55–3.88)	<0.001
*P* trend	–	<0.001

* CI, confidence interval; ILD, interstitial lung disease; IPF, idiopathic pulmonary fibrosis; RA, rheumatoid arthritis; VARA, Veterans Affairs Rheumatoid Arthritis.

^a^ Prevalent ILD estimates are odds ratios, and incident ILD estimates are hazard ratios. Models were adjusted for age, sex, race, smoking status, anti–cyclic citrullinated peptide antibody positivity, and baseline RA disease activity.

**Figure 2:**
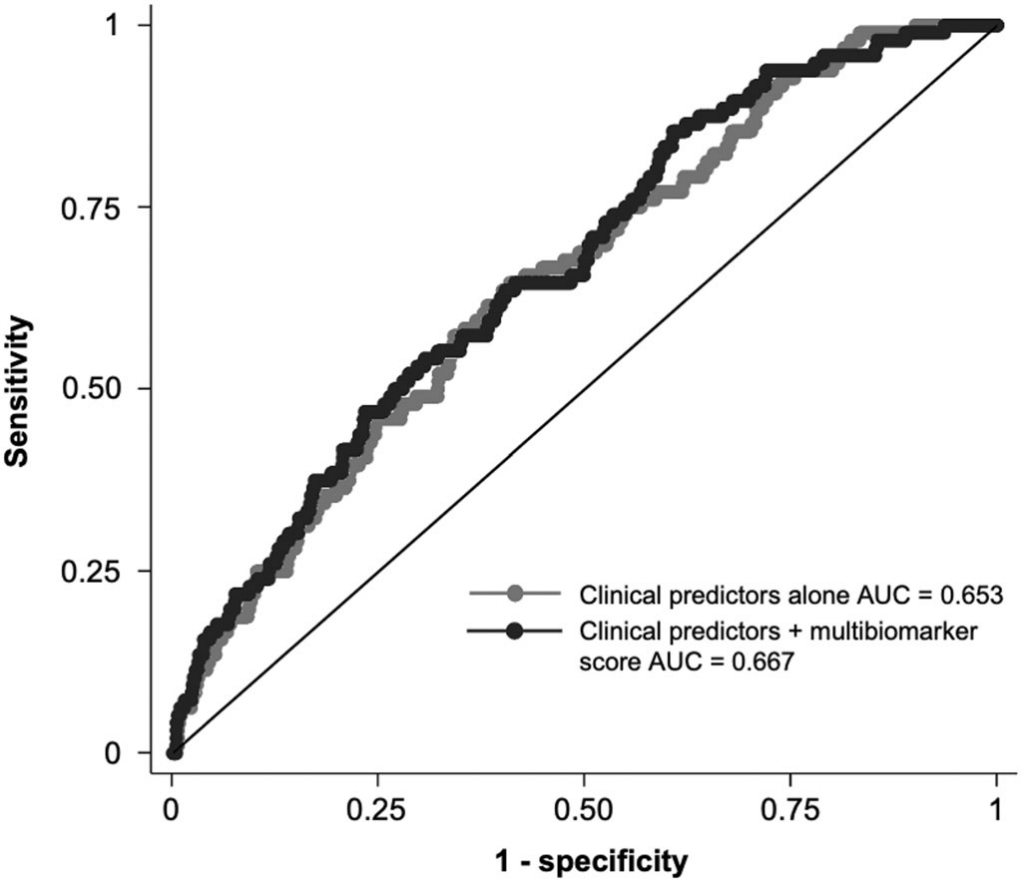
Receiver operating characteristic curves for clinical predictors versus multibiomarker score in cross‐sectional analyses of prevalent RA‐ILD (validation cohort). Clinical predictors included age, sex, race, smoking status, anti–cyclic citrullinated peptide antibody positivity, and baseline DAS28. The multibiomarker score was measured as a continuous variable. AUC, area under the curve; DAS28, Disease Activity Score in 28 joints; ILD, interstitial lung disease; RA, rheumatoid arthritis.

In ROC analyses, the addition of the IPF multibiomarker score to clinical variables alone (age, sex, race, smoking status, anti‐CCP antibody positivity, and baseline DAS28) did not significantly improve prevalent RA‐ILD discrimination (area under the curve 0.667 vs 0.653, *P* = 0.32; Figure 2). We assessed the sensitivity and specificity, as well as the positive and negative predictive values, of the multibiomarker score at varying cut points (Supplemental Table 2). Although a multibiomarker score greater than −1 had a sensitivity of 80% for RA‐ILD, the specificity was only 32%. Scores ≥4 had a specificity of ≥90% but were present in only 11% of the cohort and had a sensitivity of 17%. Overall, positive predictive values were low and negative predictive values were high, reflecting the low prevalence of ILD in the VARA cohort.

**Figure 3:**
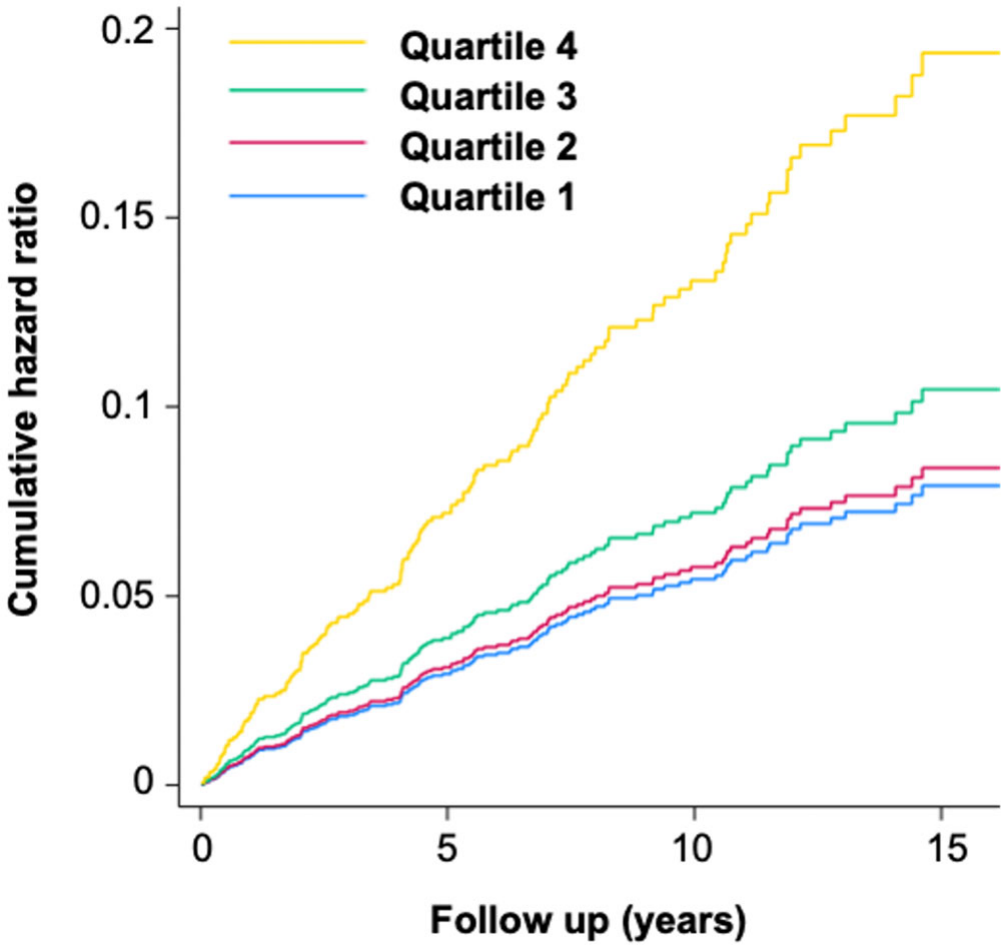
Cumulative hazard of incident RA‐ILD by multibiomarker score quartiles (validation cohort). Cumulative hazard plots were constructed using Cox models and adjusted for age, sex, race, smoking, anti–cyclic citrullinated peptide antibody positivity, RA disease activity, and period of enrollment. The x‐axis indicates follow‐up time (years), and the y‐axis indicates the cumulative hazard of incident RA‐ILD in adjusted models. ILD, interstitial lung disease; RA, rheumatoid arthritis. Color figure can be viewed in the online issue, which is available at http://onlinelibrary.wiley.com/doi/10.1002/art.43383/abstract.

Participants who developed incident ILD during longitudinal follow‐up had higher IPF multibiomarker scores at registry enrollment than those without ILD (mean 0.60 vs −0.08; *P* = 0.031). Among individual analytes in the score, IL‐8, MMP‐7, and MMP‐9 concentrations were all significantly higher in participants who developed incident ILD (Table 1). In adjusted analyses, higher multibiomarker scores were associated with a greater risk of incident ILD (adjusted hazard ratio [aHR] 1.08 [95% CI 1.03–1.13] per one‐point increase). Those in the highest quartile of IPF multibiomarker scores had a greater than two‐fold increased risk of incident ILD (aHR 2.44 [95% CI 1.55–3.88]; Table 2). Over 15 years of follow‐up, those in the highest quartile of multibiomarker scores had a cumulative hazard of incident ILD approaching 20%, compared to <10% for quartiles 1, 2, and 3 (Figure 3). Relative to clinical predictors alone, the addition of the multibiomarker score led to a significant but modest improvement in discrimination of incident RA‐ILD when divided into quartiles (Harrell's C statistic 0.627 vs 0.666, *P* = 0.028) but not as a continuous measure (Harrell's C statistic 0.627 vs 0.644, *P* = 0.29).

### Exploratory analyses in validation cohort

Among VARA participants with prevalent ILD, 55 had ILD patterns classified as UIP and 51 had ILD patterns classified as non‐UIP. As a continuous measure, associations of the IPF multibiomarker score with prevalent ILD were similar in non‐UIP (aOR 1.10 [95% CI 1.00–1.21] per one‐point increase) and UIP (aOR 1.05 [95% CI 0.97–1.14] per one‐point increase). Those in the highest quartile of multibiomarker scores had significantly higher odds of prevalent non‐UIP (aOR 3.07 [95% CI 1.20–7.83]), whereas odds of UIP did not reach significance for those in the highest quartile of multibiomarker scores (Supplemental Table 3).

Among participants who developed incident ILD, 85 had ILD patterns classified as UIP and 68 had ILD patterns classified as non‐UIP. As a continuous measure, the IPF multibiomarker score was similarly associated with incident non‐UIP (aHR 1.10 [95% CI 1.02–1.19] per one‐point increase) and UIP (aHR 1.06 [95% CI 1.00–1.13] per one‐point increase). Those in the highest quartile of multibiomarker scores had a >2.5‐fold increased risk of incident ILD, with associations observed for both non‐UIP and UIP patterns (Supplemental Table 3).

### Sensitivity analyses in validation cohort

When the 50 patients with overlapping data from the validation and development cohorts were excluded from analysis of the VARA cohort, associations of the IPF multibiomarker score (continuous and quartiles) with prevalent and incident ILD were similar in magnitude and significance (Supplemental Table 4) to parallel analyses including these individuals (Table 2). In sensitivity analyses excluding MMP‐7 (which has previously been linked to prevalent and incident RA‐ILD in this cohort), the IPF multibiomarker score was modestly attenuated and no longer significantly associated with prevalent ILD as a continuous measure (aOR 1.04 [95% CI 0.97–1.11] per one‐point increase), and similarly the highest quartile multibiomarker score was no longer significantly associated with prevalent ILD (aOR 1.66 [95% CI 0.92–3.00]). In contrast, although results were attenuated relative to the primary analysis, the IPF multibiomarker score remained significantly associated with incident ILD after excluding MMP‐7 (aHR 1.06 [95% CI 1.01–1.11] per one‐point increase; aHR 2.00 [95% CI 1.26–3.17] for the highest quartile). Following reclassification of ILD identified within the first year after registry enrollment as prevalent ILD (n = 132) and ILD identified more than one year after registry enrollment as incident ILD (n = 127), associations of the IPF multibiomarker score with ILD were similar to those observed in the primary analyses, both as a continuous measure (aOR 1.06 [95% CI 1.00–1.12] per one‐point increase for prevalent ILD, aHR 1.10 [95% CI 1.04–1.16] per one‐point increase for incident ILD) and by quartile (data not shown).

## DISCUSSION

In this observational study with independent development and validation cohorts, we found that a panel of IPF‐derived peripheral biomarkers was similarly associated with prevalent and incident ILD among participants with RA. These findings were strongest for the development of incident RA‐ILD, as those with the highest quartile of multibiomarker scores demonstrated a nearly 20% risk of developing incident RA‐ILD over 15 years of longitudinal follow‐up. Moreover, these results persisted when analyzing UIP and non‐UIP RA‐ILD separately. Together, these findings provide further support for a shared etiopathogenesis between RA‐ILD and IPF and illustrate the potential value of peripheral blood biomarker panels for RA‐ILD risk stratification.

There exists an unmet need to accurately identify patients with RA at an increased risk for the presence or development of ILD, and routinely measured clinical and laboratory data have a limited ability to fill this void.^7^ Accurate measures to identify these patients may help to select patients with RA who are more likely to benefit from earlier or more frequent screening for RA‐ILD with chest HRCT and pulmonary function tests. The measurement of peripheral blood biomarkers is an attractive, minimally invasive approach with the potential for widespread application to help bridge this gap. In this study, we found that a multibiomarker score of seven IPF‐derived peripheral blood biomarkers was associated with both prevalent and incident RA‐ILD risk. Even when removing the known RA‐ILD–associated biomarker MMP‐7 from the panel,^15^ the IPF multibiomarker score remained significantly associated with incident, but not prevalent, RA‐ILD.

Patients with RA with the highest quartile of IPF multibiomarker scores had a greater than two‐fold increased risk of prevalent and incident RA‐ILD, although the multibiomarker score did not demonstrate sufficient clinical utility for the identification of prevalent ILD. Model performance metrics (eg, area under the ROC curve) did not show improved discrimination over clinical risk factors alone, and none of the cut points demonstrated sufficient sensitivity and specificity. These results exemplify the challenge of constructing a clinically meaningful biomarker panel that is associated with ILD risk. Further research is needed to identify the clinical, laboratory, and genetic risk factors that can accurately identify patients with RA with or at risk for ILD. An additional unmet need is a strategy to identify patients with RA with subclinical ILD at greater risk to progress to clinically apparent RA‐ILD, thus potentially warranting closer monitoring or earlier intervention. In the development cohorts, there was no statistically significant difference in multibiomarker scores between patients with RA without ILD and those with subclinical ILD. However, there was a numerical increase in the mean multibiomarker score across higher stages of disease (no ILD vs subclinical ILD vs clinically apparent ILD). Moreover, the statistically significant association of the IPF multibiomarker panel with the development of incident ILD in the VARA validation cohort suggests that this panel may have clinical utility in identifying at‐risk individuals without ILD or with subclinical ILD who would benefit from heightened surveillance for emergence of clinically significant ILD. Based on our findings, future studies with large sample sizes and comprehensive phenotyping of subclinical ILD status are clearly needed to more adequately address this question.

Although this multibiomarker score does not add significantly to established RA‐ILD risk factors for the predictive value of RA‐ILD, the observed associations with prevalent and incident RA‐ILD do lend further support to the existence of common final pathways linking RA‐ILD and IPF. RA‐ILD shares a number of risk factors, histopathologic and radiologic findings, and clinical features with IPF, suggesting an overlap in the etiopathogenesis of these conditions.^6^ Recent genetic studies finding the *MUC5B* promoter variant, the strongest genetic risk factor for IPF, to also be associated with RA‐ILD in a UIP pattern further support this connection.^16^, ^17^ In this study, we have added to the growing body of literature by observing associations of IPF‐derived peripheral blood biomarkers (eotaxin, Flt‐3L, IL‐8, MDC, MCP‐1, and MMP‐2/7/9) with prevalent and incident RA‐ILD. This observation suggests that similar processes may be occurring on a molecular level in IPF and RA‐ILD. Importantly, this potential molecular overlap raises the possibility that treatment responses to pharmacologic therapies could be shared between IPF and some patients with RA‐ILD. Indeed, recent studies have suggested that antifibrotic treatments used for IPF may similarly slow the rate of decline in lung function in patients with RA‐ILD.^18^, ^19^ Given the limited body of evidence for effective immunomodulatory therapy in RA‐ILD and the general avoidance of immunomodulatory therapy in IPF,^20^, ^21^ it is unknown whether selected molecular “endotypes” may define subsets of RA‐ILD and/or IPF with greater response to immunomodulatory therapy.

A recent meta‐analysis including studies of RA‐ILD reported UIP as the most common pattern of RA‐ILD, followed by nonspecific interstitial pneumonia.^22^ Consistent with this finding, we observed a slightly higher proportion of UIP‐ILD in this study, based on the clinical radiology read. Because UIP is the pattern of IPF, we performed secondary analyses evaluating the performance of the IPF‐associated multibiomarker score in patients with UIP versus non‐UIP RA‐ILD. In incident ILD analyses, the multibiomarker score was associated with both a UIP and a non‐UIP RA‐ILD pattern. However, in prevalent ILD analyses, associations were stronger for the non‐UIP pattern. This finding was unexpected. Given the smaller sample sizes of this secondary analysis and potential misclassification of ILD pattern based on clinical radiology reads, our findings should be considered hypothesis generating. Of note, previous research in this cohort did show an association of the *MUC5B* rs35705950 promoter variant (an established risk factor for IPF and UIP RA‐ILD) with UIP RA‐ILD but not non‐UIP RA‐ILD,^16^ suggesting validity of the case definitions for UIP RA‐ILD and non‐UIP RA‐ILD. It is possible non‐UIP RA‐ILD may later evolve into UIP RA‐ILD.^23^ In this situation, the molecular pathways leading to UIP could be highly activated in advance of characteristic radiographic abnormalities indicative of UIP. Future studies with longitudinal, comprehensive phenotyping of RA‐ILD subtypes are warranted to better understand any potential differences in biomarker profiles that may be present among different clinical, radiographic, and/or histopathologic subtypes.

Although we did not evaluate the progression of radiologic or physiologic ILD parameters in this study, future studies should also evaluate whether levels of these IPF‐associated proteins, or other peripheral blood biomarkers, are able to meaningfully predict RA‐ILD progression. Not all patients with RA‐ILD appear to show substantial progression over time,^24^, ^25^, ^26^ and the identification of patients at the greatest risk for progression may help to identify patients most likely to benefit from immunosuppressive or antifibrotic therapies. Similarly, we were not able to assess whether biomarker measurements differ among patients with early versus established RA‐ILD in the validation cohort, which will require studies with longitudinal clinical follow‐up and serial biomarkers measurements.

There are limitations to this study. The validation cohort used was a US veteran population with a large proportion of older men who were current or former smokers, which may impact generalizability. However, this is a useful population to study because it represents a high‐risk group for the development of RA‐ILD, and findings from the non‐VA development cohort suggest that similar associations may be seen in other cohorts with more representative clinical/demographic profiles. There were also minor differences in the analytes included in the IPF multibiomarker scores between the development and validation cohorts. Despite these differences, the results consistently showed an association of a composite IPF‐derived peripheral biomarker score with RA‐ILD. Different methods were used to define the presence of RA‐ILD in the development and validation cohorts, but the reproducible results across cohorts emphasize the consistency and generalizability of our findings. Finally, there was partial overlap in participants from the VA development cohort and the VARA validation cohort (n = 50); importantly, however, excluding these participants in a sensitivity analysis did not meaningfully change results.

In conclusion, this study showed that an IPF‐derived peripheral blood biomarker panel was associated with RA‐ILD in multiple independent cohorts. These findings provide additional evidence supporting a shared etiopathogenesis of RA‐ILD and IPF. However, the current multibiomarker score did not demonstrate sufficient performance metrics for clinical utility. Future studies incorporating additional peripheral blood biomarkers, agnostic biomarker analyses, and other clinical measures are needed to develop and validate clinically meaningful tools to stratify ILD risk for patients with RA.

## AUTHOR CONTRIBUTIONS

All authors contributed to at least one of the following manuscript preparation roles: conceptualization AND/OR methodology, software, investigation, formal analysis, data curation, visualization, and validation AND drafting or reviewing/editing the final draft. As corresponding author, Dr. England confirms that all authors have provided the final approval of the version to be published and takes responsibility for the affirmations regarding article submission (eg, not under consideration by another journal), the integrity of the data presented, and the statements regarding compliance with institutional review board/Declaration of Helsinki requirements.

## Supporting information


**Disclosure form**.

**Appendix S1:** Supplementary Information.
