# Supplementary material for: Performance of an Idiopathic Pulmonary Fibrosis–Derived Multibiomarker Panel for Rheumatoid Arthritis–Associated Interstitial Lung Disease
Source: Arthritis Rheumatol. 2026 Jan 11;78(2):302–10. doi: 10.1002/art.43383 (PMC12936895; doi:10.1002/art.43383)
Supplement: Supplementary file 2 — Appendix S1: Supplementary Information. [file ART-78-302-s001.docx]

**SUPPLEMENTAL MATERIAL**

Performance of an Idiopathic Pulmonary Fibrosis Derived Multibiomarker Panel for Rheumatoid Arthritis-Associated Interstitial Lung Disease

Luedders BA et al.

**Supplemental Table 1.** Baseline characteristics of VA and non-VA development cohorts.

**Supplemental Table 2**. Test performance of IPF multibiomarker score for prevalent RA-ILD at varying cutoffs (validation cohort).

**Supplemental Table 3.** Association of IPF multibiomarker score with prevalent and incident RA-ILD stratified by ILD pattern (validation cohort).

**Supplemental Table 4.** Association of IPF multibiomarker score with prevalent and incident RA-ILD in validation cohort excluding overlapping patients.

**Supplemental Table 1.** Baseline characteristics of VA and non-VA development cohorts.

|  | **VA RA-ILD** | **VA RA-no-ILD** | **Non-VA RA-ILD** | **Non-VA RA-no-ILD** |
| --- | --- | --- | --- | --- |
| n | 76 | 17 | 49 | 22 |
| Age, years | 65.0 (9.9) | 53.0 (13.1) | 65.2 (10.8) | 50.3 (14.2) |
| Male sex, %* | 94.7 | 64.7 | 36.7 | 23.8 |
| White race, %* | 68.4 | 64.7 | 75.5 | 71.4 |
| Smoking status, ever, %* | 88.2 | 41.2 | 55.1 | 42.1 |
| RA duration, years* | 11.5 (10.0) | 12.0 (10.0) | 12.8 (10.3) | 8.3 (9.4) |
| Anti-CCP positive, % | 92.1 | 70.6 | 85.7 | 81.8 |
| DAS28* | 3.77 (1.31) | 3.76 (0.95) | 3.68 (1.14) | 3.41 (1.33) |
| Values mean (SD) or %  *Missing data: sex (n=1 non-VA RA-no-ILD), race (n=1 non-VA RA-no-ILD), smoking status (n=1 non-VA RA-no-ILD), RA duration (n=5 VA RA-ILD, n=4 VA RA-no-ILD, n=1 non-VA RA-no-ILD), DAS28 (n= 8 non-VA RA-ILD, n=6 non-VA RA-no-ILD)  Abbreviations: RA = rheumatoid arthritis, ILD = interstitial lung disease, CCP = cyclic citrullinated peptide, DAS28 = 28-joint disease activity score | | | | |

**Supplemental Table 2**. Test performance of IPF multibiomarker score for prevalent RA-ILD at varying cutoffs (validation cohort).

| **Cut-point*** | | **Sensitivity (%)** | | **Specificity (%)** | | **PPV (%)** | | **NPV (%)** | | **Percentage above^x^ (%)** |
| --- | --- | --- | --- | --- | --- | --- | --- | --- | --- | --- |
| -12 | 100 | | 0.58 | | 5.59 | | 100 | | 99.45 | |
| -11 | 99.24 | | 1.56 | | 5.61 | | 97.22 | | 98.48 | |
| -10 | 98.48 | | 2.99 | | 5.64 | | 97.10 | | 97.09 | |
| -9 | 96.97 | | 4.11 | | 5.62 | | 95.83 | | 95.95 | |
| -8 | 92.42 | | 5.67 | | 5.46 | | 92.70 | | 94.23 | |
| -7 | 90.15 | | 7.45 | | 5.43 | | 92.78 | | 92.41 | |
| -6 | 88.64 | | 8.48 | | 5.40 | | 92.68 | | 91.36 | |
| -5 | 86.36 | | 9.86 | | 5.34 | | 92.47 | | 89.93 | |
| -4 | 85.61 | | 12.41 | | 5.44 | | 93.60 | | 87.48 | |
| -3 | 84.85 | | 16.51 | | 5.65 | | 94.87 | | 83.56 | |
| -2 | 83.33 | | 22.98 | | 5.99 | | 95.90 | | 77.37 | |
| -1 | 80.30 | | 32.22 | | 6.52 | | 96.52 | | 68.48 | |
| 0 | 71.97 | | 43.78 | | 7.01 | | 96.37 | | 57.10 | |
| 1 | 61.36 | | 56.72 | | 7.71 | | 96.14 | | 44.29 | |
| 2 | 46.97 | | 69.66 | | 8.36 | | 95.71 | | 31.27 | |
| 3 | 29.55 | | 81.26 | | 8.50 | | 95.14 | | 19.34 | |
| 4 | 17.42 | | 89.69 | | 9.06 | | 94.86 | | 10.70 | |
| 5 | 7.58 | | 95.23 | | 8.55 | | 94.59 | | 4.93 | |
| 6 | 4.55 | | 98.26 | | 13.33 | | 94.59 | | 1.89 | |
| 7 | 2.27 | | 99.38 | | 17.65 | | 94.52 | | 0.72 | |
| 8 | 1.52 | | 99.87 | | 40.00 | | 94.51 | | 0.21 | |
| *Any score above the cut-point considered a positive test.  ^x^Percentage of patients with multibiomarker scores greater than cut-point  Abbreviations: PPV = positive predictive value, NPV = negative predictive value | | | | | | | | | | |

**Supplemental Table 3.** Association of IPF multibiomarker score with prevalent and incident RA-ILD stratified by ILD pattern (validation cohort).

| **Quartile** | **Adjusted odds (prevalent) or hazard (incident) ratio (95% CI)** | **p-value** |
| --- | --- | --- |
| ***UIP-ILD*** | | |
| *Prevalent* | | |
| Quartile 1 | Ref. |  |
| Quartile 2 | 0.71 (0.28, 1.79) | 0.47 |
| Quartile 3 | 0.97 (0.42, 2.24) | 0.94 |
| Quartile 4 | 1.62 (0.76, 3.47) | 0.22 |
| P trend |  | 0.21 |
| *Incident* | | |
| Quartile 1 | Ref. |  |
| Quartile 2 | 1.00 (0.49, 2.06) | 1.00 |
| Quartile 3 | 1.23 (0.62, 2.45) | 0.56 |
| Quartile 4 | 2.54 (1.36, 4.72) | 0.003 |
| P trend |  | 0.025 |
| ***Non-UIP-ILD*** | | |
| *Prevalent* | | |
| Quartile 1 | Ref. |  |
| Quartile 2 | 0.81 (0.25, 2.69) | 0.73 |
| Quartile 3 | 2.15 (0.81, 5.69) | 0.12 |
| Quartile 4 | 3.07 (1.20, 7.83) | 0.019 |
| P trend |  | 0.018 |
| *Incident* | | |
| Quartile 1 | Ref. |  |
| Quartile 2 | 1.14 (0.53, 2.43) | 0.74 |
| Quartile 3 | 1.24 (0.58, 2.65) | 0.58 |
| Quartile 4 | 2.53 (1.29, 4.98) | 0.007 |
| P trend |  | 0.015 |
| Models adjusted for age, sex, race, smoking status, anti-citrullinated peptide antibody positivity, and baseline RA disease activity.  Abbreviations: UIP = usual interstitial pneumonia, ILD = interstitial lung disease | | |

**Supplemental Table 4.** Association of IPF multibiomarker score with prevalent and incident RA-ILD in validation cohort excluding overlapping patients.

| **Quartile** | **Adjusted odds or hazard ratio (95% CI)*** | **p-value** |
| --- | --- | --- |
| *Prevalent ILD* | | |
| Quartile 1 | Ref. |  |
| Quartile 2 | 0.89 (0.39, 2.05) | 0.79 |
| Quartile 3 | 1.37 (0.65, 2.89) | 0.42 |
| Quartile 4 | 2.60 (1.31, 5.16) | 0.006 |
| P trend |  | 0.004 |
| *Incident ILD* |  |  |
| Quartile 1 | Ref. |  |
| Quartile 2 | 1.06 (0.62, 1.82) | 0.83 |
| Quartile 3 | 1.33 (0.79, 2.25) | 0.28 |
| Quartile 4 | 2.36 (1.46, 3.82) | <0.001 |
| P trend |  | <0.001 |
| *Prevalent ILD estimates are odds ratios while incident ILD estimates are hazard ratios.  Models adjusted for age, sex, race, smoking status, anti-citrullinated peptide antibody positivity, and baseline RA disease activity.  Abbreviations: ILD = interstitial lung disease | | |
